# A test of memory for stimulus sequences in great apes

**DOI:** 10.1371/journal.pone.0290546

**Published:** 2023-09-06

**Authors:** Johan Lind, Vera Vinken, Markus Jonsson, Stefano Ghirlanda, Magnus Enquist

**Affiliations:** 1 Centre for Cultural Evolution, Stockholm University, Stockholm, Sweden; 2 Biosciences Institute, Newcastle University, Newcastle upon Tyne, United Kingdom; 3 Department of Psychology, CUNY Graduate Center, New York, NY, United States of America; 4 Department of Psychology, Brooklyn College, New York, NY, United States of America; 5 Department of Zoology, Stockholm University, Stockholm, Sweden; National University of Singapore, SINGAPORE

## Abstract

Identifying cognitive capacities underlying the human evolutionary transition is challenging, and many hypotheses exist for what makes humans capable of, for example, producing and understanding language, preparing meals, and having culture on a grand scale. Instead of describing processes whereby information is processed, recent studies have suggested that there are key differences between humans and other animals in how information is recognized and remembered. Such constraints may act as a bottleneck for subsequent information processing and behavior, proving important for understanding differences between humans and other animals. We briefly discuss different sequential aspects of cognition and behavior and the importance of distinguishing between simultaneous and sequential input, and conclude that explicit tests on non-human great apes have been lacking. Here, we test the memory for stimulus sequences-hypothesis by carrying out three tests on bonobos and one test on humans. Our results show that bonobos’ general working memory decays rapidly and that they fail to learn the difference between the order of two stimuli even after more than 2,000 trials, corroborating earlier findings in other animals. However, as expected, humans solve the same sequence discrimination almost immediately. The explicit test on whether bonobos represent stimulus sequences as an unstructured collection of memory traces was not informative as no differences were found between responses to the different probe tests. However, overall, this first empirical study of sequence discrimination on non-human great apes supports the idea that non-human animals, including the closest relatives to humans, lack a memory for stimulus sequences. This may be an ability that sets humans apart from other animals and could be one reason behind the origin of human culture.

## Introduction

Understanding why non-human animals do not learn languages, mental arithmetic, or have culture on a grand scale, is a challenge [1–6]. Many attempts have been aimed at identifying minimal cognitive differences between humans and other animals by focusing on how animals process already acquired information. Some debated suggestions concern whether humans alone understand grammar [7–11], engage in causal learning [12–15], represent higher-order relationships [16, 17], or plan for the future [18–22]. Here, we address this challenge instead by focusing on the ability to faithfully recognize and represent arbitrary sequential information, that is, how an organism represent multiple arbitrary stimuli that are separated in time [23].

### A few comments on sequential aspects of cognition and behavior

Before delving into details on memory for stimulus sequences, we start with a few brief comments on the sequential aspects of cognition and behavior that are explored in this study.

First, our study concerns temporal sequences (see [23] for details). It is not about single events, or simultaneous presentations of more than one stimulus, such as compound stimuli. One could argue that both sensory input and behavioral output are by necessity stretched out in time and can therefore, in some sense, be described as having sequential properties, like the sound of a whistle or when a piece of food is swallowed. However, from the early ethologists (e.g. [24]), comparative psychologists (e.g. [25]), and behaviorists (e.g. [26]), stimuli and behaviors have been analysed and categorized as meaningful and identifiable events by using coarse grained enough time scales and functional analyses of stimuli and behavior. This way, hearing a whistle, or swallowing a piece of food, can be described as two different single events. By the same reasoning, two stimuli that are presented on a screen, one after the other, can be treated as a sequence consisting of two identifiable events.

Second, our study concerns recognizing and remembering input (stimuli) reaching an organism. It does not concern behavioral output. This is important because performing a behavior sequence does not *per se* require a representation of a stimulus sequences. In reinforcement learning, behavior sequences can be performed without a memory of the whole learned sequence [27, 28] (faithful sequence memories can of course be implemented in artificial intelligence systems, by for example storing all moves in memory during a chess game [29]). Instead, behaviors in the sequence are learned individually and linked through primary and conditioned reinforcement. This kind of linking individual behaviors to form complex sequences of behavior forms the basis of training behavioral chains in the experimental analysis of behavior, in behavioral psychology, and in applied behavior analysis [30–32]. Furthermore, computational models of animal learning can account for the development of behavior sequences without a need for faithful representations of stimulus sequences ([33], see also [34], and chapter 3 and 5 in Enquist et al. 2023 [6], for in-depth discussions of sequential behavior and memory for stimulus sequences). Such learning models have been shown to reproduce well-established learning phenomena in experimental psychology [35, 36], and provide plausible accounts for how various behavior sequences can be acquired in non-human animals (e.g. in tool use: [33], planning behavior: [21], social learning: [37], and caching behavior [38].

To conclude, here we focus on how animals recognize and remember stimulus sequences, that is temporal series of at least two successive stimuli. We do not focus on how animals represent single stimuli, or many stimuli that are presented simultaneously. For these reasons, test paradigms that involve simultaneously presented arrays of stimuli are beyond the scope of this study [39, 40], as responding to simultaneous input does not require the recognition of temporal stimulus sequences, even if subjects perform behavior sequences in response to complex input [41]. This also applies to the well-known studies where chimpanzees learned to point to the location of up to nine numerals that were presented simultaneously (see [42, 43] for studies on chimpanzees, and [44–46] for further discussion about these results). For the same reason we do not focus on how animals learn behavior sequences. Therefore, how animals use various tools [47–49], and perform other behavior sequences when communicating with sounds or gestures [50–53] is also beyond the scope of this study.

Ideas about how cognitive bottlenecks can constrain cognitive processes and behavior make it interesting to study the recognition and memory of stimulus sequences. This is because how sequential information is represented and remembered may affect subsequent cognitive processes and expected behavioral outcomes. If, for example, an aspect of information is lost already when it is perceived, it can neither be accessed nor used in later processes to affect behavior. In other words, mechanisms for the acquisition and representation of information can act as bottlenecks for subsequent representational levels and behavior [54].

### Memory for stimulus sequences

A well established finding of a tentative cognitive bottleneck, rarely considered in contemporary discussions on animal cognition, comes from delayed matching-to-sample studies (see early work, e.g. [55–57], and text books, e.g. [58, 59]). In such studies, a single stimulus is typically presented to an animal for a few seconds. The disappearance of the stimulus marks the onset of a delay, and after the delay two stimuli are presented together. One of these stimuli is identical to the sample, whereas the other stimulus is different, and the animal is rewarded for choosing the identical stimulus matching the sample. Hundreds of delayed matching-to-sample studies have been performed on birds and non-human mammals showing that animals will learn this task to near perfection with zero-second delays, given sufficient training. However, performance of all non-human species degrade rather quickly, even after short delays. Humans can, on the other hand, easily reach error-free performance with delays of 48 hours [6, 60]. These differences in a general-purpose memory system, that birds and non-human mammals quickly forget arbitrary stimuli, whereas humans can retain such information for days and weeks [61], have been put forward as a tentative cause for the observed cognitive and cultural divide between humans and other animals [6, 34, 46, 62].

Furthermore, the general pattern of rapid memory decay of single stimuli matches results on how animals recognize and remember sequences of stimuli [23]. The common denominator is that patterns of animal working memory adhere to the idea of “trace memory” representation, which means that representations of single stimuli have no definite duration and fade with time [63–65]. If an animal sees a green light followed by a red light, it will at a subsequent time step have a stronger representation of the red light, as the memory of the green light has faded more because it was observed before the red light. Ghirlanda et al. [23] analyzed over 100 stimulus sequence discrimination experiments (from 14 bird and mammal species). The study included data from various test paradigms where animals have been subjected to temporal sequences of stimuli, including, for instance, rule learning [66, 67], artificial grammar studies [7, 68, 69], sequence discriminations [70, 71], song recognition in birds [72, 73], and some clinical studies [74, 75]. The analysis found systematic and pervasive errors as expected from a trace memory model, irrespective of the origin of the data. The study showed that animals confuse, for example, a red-green sequence of lights with green-red and green-green sequences, and that these kinds of errors persist after thousands of learning trials. Just like in delayed matching-to-sample studies, no systematic differences were found between species due to ecological niches or evolutionary history. In contrast, humans tell sequences apart nearly immediately, represent the order of stimuli faithfully, and have no difficulty discriminating between red-green vs. green-red sequences of lights [23]. Thus, it was concluded that representing sequential information faithfully sets humans apart from other animals, with one crucial caveat: no explicit tests have been performed to determine if non-human great apes represent stimulus sequences as unstructured collections of memory traces, as was found in other non-human animals. There are studies where chimpanzees have been subjected to stimulus sequences [76, 77], but these studies were not designed to test alternative hypotheses, for example if responses can be explained by trace memory representations [23].

To test the hypothesis that humans alone have evolved a capacity to faithfully represent sequences of stimuli we here report the first comparative tests on humans and another species of great apes, bonobos (*Pan paniscus*). Bonobos are together with chimpanzees the closest extant relatives to humans. Our aim was to test if the closest relative to humans also represents sequential information faithfully, or confuses even short sequences of stimuli as other non-human animals do. First, we tested bonobos’ memory for single stimuli to see if they, like all other tested non-human mammals and birds [60], rapidly forget an arbitrary stimulus. Second, we tested if bonobos can tell the difference between two stimulus sequences, to see if bonobos like all other tested animals find it exceedingly difficult to tell even short sequences apart. Although we know that many human abilities rely upon memory for stimulus sequences and require exact representation of order [23], we included a sequence discrimination test on human subjects that mirrored the test for bonobos for the sake of a direct comparison of results between humans and bonobos. Finally, we subjected bonobos to an explicit test of the memory trace model previously described [23].

## Materials and methods

All bonobos were born and raised in captivity and housed at the Ape Initiative (the former Ape Cognition and Conservation Initiative) in Des Moines, Iowa, USA. For experiments on human subjects, five students at Brooklyn College, New York, USA, took part in the study.

### Ethical statement

Participation in all sessions was voluntary and a bonobo could at any time interrupt and leave a test session. In addition, for bonobos, daily sessions were limited to a maximum of 120 trials per individual. Procedures of these tests comply with the ASAB/ABS Guidelines for the Use of Animals in Research and the study was approved by the Institutional Animal Care and Use Committee of the Ape Cognition and Conservation Initiative (IACUC #190203–01 and #190203–02).

For the experiment on humans, all participants were recruited and took part in this non-invasive study on a voluntary basis. Subjects were allowed to leave at any time. These experiments were authorised by Brooklyn College Institutional Review Board (IRB), and only SG had access to information that could identify individual participants during the study.

### Common procedures

All tests were performed using automated computer-controlled screens, and all subjects were used to screens. Bonobos were experienced touch-screen users and students were experienced computer-mouse users. Behavioral data was recorded automatically as a screen was touched, or in the human part when the mouse was clicked. During rewarding trials, the computer program elicited one of two distinct sounds after each response. In these trials, correct responses were followed by a chiming sound. In tests on bonobos, this cued the experimenter to deliver a food reward (rewards were generally of high value, most commonly grapes, peanuts, and strawberries were used). In tests on humans, a large happy face-emoji appeared on the screen simultaneously as this sound played. Furthermore, after an incorrect response, a buzzing sound was played and the screen turned black. For bonobos, no subsequent food reward was delivered, and for humans, a large sad face-emoji appeared together with the buzzing sound. Bonobos were only tested when alone in their testing environment, with one exception. In one session, Kanzi requested to work on the touchscreen, but Maisha did not want to leave that enclosure. As the two apes did not interact in any way, Kanzi did not show any sign of distraction, and they were in opposite parts of the room, we decided to allow Kanzi to perform tests despite Maisha’s presence.

Inadvertent cueing of bonobos’ responses was avoided in one of two ways. When using a dedicated test room, the experimenter was out of sight when the bonobo was facing the touchscreen, and when tested in another enclosure, the experimenter could not see the touchscreen the apes were working on.

A daily session began with a pause screen. If the ape pressed the screen during a pause, the program did not respond. The experimenter started the program by using the keyboard and every trial started with the presentation of a *next button*, a large +-shaped button. Different test stimuli were used in the different parts of our study. All programs, including audio- and picture files, can be found at https://github.com/markusrobertjonsson/bonobo/tree/269e94e.

For bonobos, we started with delayed matching-to-sample tests and continued with sequence discrimination tests. These parts were performed during 2019. We performed trace model tests during spring 2022. Human sequence discriminations were performed during fall 2019.

### Apparatus

At the Ape Initiative, bonobos interacted with the programs through touchscreens (a 24” Elo Touch touchscreen (ET2401LM-8CWA) connected to a HP Pavilion Laptop, and a 32” Elo Touch touchscreen (ET4243L) connected to a Mini Mac (A1347), refresh rate and resolution of both screens were: 60 Hz, 1920 by 1080 pixels, respectively). Human subjects were tested on a standard desktop computer, and interacted with the screen using a standard computer mouse.

### Delayed matching-to-sample in bonobos

In this study, two male bonobos, Kanzi (39 years old) and Teco (9 years old), participated.

Pre-training included both simultaneous- and zero-delay matching-to-sample. For simultaneous matching-to-sample, a sample stimulus (*A* or *B*) was first presented as a trial started, and *A* and *B* were conspicuous blue and yellow squares, respectively. The sample stimulus remained on the screen when the two response stimuli appeared, one matching- and one non-matching stimulus. Here, and in the other matching-to-sample tests, choosing the matching stimulus resulted in a food reward. The colour of the sample stimulus and the placement of the correct response stimulus (right or left) was randomized with equal representation over 10 trials. Task performance was measured as the frequency of correct trials over the last 20 trials performed (within a session). After having reached a criterion of 80% correct choices over the last 20 trials, training of zero-delay matching-to-sample began. The zero-delay matching-to-sample was similar to the above, but the sample stimulus was presented for 2 seconds at the beginning of the trial before it disappeared. Upon disappearance, the two response stimuli appeared without delay and a response to the matching stimulus was rewarded. After having reached the criterion of 80% correct choices over the last 20 trials, the delayed matching-to-sample test started.

In the delayed matching-to-sample test we introduced delays between the disappearance of the sample stimulus and the appearance of the two response stimuli. Delay durations were 0, 2, 5, and 10 seconds. Every second trial was a 0-second delay to keep reward rates high, and the delays of the remaining trials were determined in a pseudo-random order. For this reason, subjects experienced more 0-second delays than other delays. And Kanzi experienced more 5-second delays than Teco because Kanzi started the delayed matching-to-sample with 5-second delays, and we subsequently introduced other delays as no clear signs of improvements were found. Performance was measured as the frequency of correct trials over the last 20 trials performed.

### Sequence discrimination in bonobos

Here, four male bonobos (Kanzi, Nyota, Maisha and Teco, age range: 9–39 years old) participated. This test was inspired by the two-event sequence discrimination study on pigeons performed by Weisman et al. [70]. Instead of using a go-no-go paradigm, we used a two-choice paradigm with two response buttons: a left button (with horizontal bars), and a right button (with vertical bars). During tests, two stimuli were presented following each other, each stimulus was present on the screen for 1 second, and the inter-stimulus interval was 300 milliseconds. Response buttons appeared when the last stimulus of the sequence disappeared.

With two stimuli, *A* and *B*, the sequences *AB*, *BA*, *AA* and *BB* can be formed. In all trials following the sequence *AB*, the rewarded response was pressing a button on the left side of the screen. Following sequences *AA*, *BA* and *BB*, pressing a response button on the right side of the screen was rewarded. To prevent bonobos from developing a side bias, sequences were presented in blocks of 18 trials, consisting of 9 *AB* trials, and three each of the *AA*, *BA*, and *BB* sequences. The order of stimulus sequences presented was randomized within these blocks. The screen turned black for 3 seconds after an incorrect answer. Instead of using blue and yellow squares as stimuli, we now used blue and yellow full screens as stimuli. Half of the subjects had ‘yellow’ as the *A*-stimulus and ‘blue’ as the *B*-stimulus, whereas the other half of the subjects had ‘blue’ as the *A*-stimulus and ‘yellow’ as the *B*-stimulus.

In these sequence discrimination experiments we used ‘correction trials’. When a subject made an incorrect choice the same sequence was repeated until they pressed the correct response button. Correction trials were included when calculating the performance of the apes. Here, we did not include a learning criterion because the aim was to quantify the acquisition of their discrimination between different stimulus sequences. All four subjects did a minimum of 2,300 trials on this task and performance was measured as the proportion of correct trials within blocks of 120 trials.

### Sequence discrimination in humans

Human participants were recruited during autumn 2019 and participation resulted in earned course credit. Authors other than SG had no access to information that could identify individual participants during or after data collection. The human test started with a single stimulus discrimination and ended with a two stimuli sequence discrimination, and all human participants performed the whole experiment within one session that lasted approximately 30 minutes. Apart from the differences noted below, the sequence discrimination was identical to the one bonobos were subjected to.

Stimulus *A* and *B* were blue and orange squares, and choice buttons were a white circle and a black triangle. In contrast to the experiments on bonobos, this human part of the study did not include food rewards. Instead of food, correct trials were followed by happy faces. A written instruction told the human subjects to get as many happy faces as possible, and as few sad faces as possible. Instructions were kept to a minimum and lacked information about the study to not give students more information about the test than what the bonobos received. Full instructions were as follows:

PLEASE READ THESE INSTRUCTIONS CAREFULLY—In this experiment, you will see different shapes on screen. Touching some shapes will make a happy face appear, touching other shapes will make a sad face appear. Your task is to get as many happy faces as possible and as few sad faces. THE TWO PARTICIPANTS WITH THE HIGHER SCORE (HAPPY FACES MINUS SAD FACES) WILL RECEIVE A $25 STARBUCKS GIFT CARD. Make sure to leave your SONA ID to enter the contest. You may interrupt the experiment at any time without penalty. If you interrupt, you will still receive course credit, but you will not enter the gift card contest. Please press the space bar to start.

After each response the same “correct”, or “incorrect”, sound was played as in the bonobo study. Presentations of the happy face lasted two seconds and the sad face continued for 5 seconds after which a blackout screen was presented. This test ended when a student reached 80% correct responses over the last 20 trials.

### Trace model test in bonobos

Here, two male bonobos (Kanzi and Teco) participated. To explicitly test if bonobos, like other non-human animals, represent stimuli as unstructured collections of memory traces [23], we used a modified zero-delay matching-to-sample test with probe trials containing stimulus sequences. Bonobos first learned a matching-to-sample task with a pool of 20 test stimuli. These stimuli were illustrations of common objects, for example illustrations of a tree, a bicycle, and hands, respectively (see supplementary information for the appearance of all stimuli). Here, a randomly drawn test stimulus was presented as a sample stimulus for 1 second. When the sample stimulus disappeared, four stimuli appeared on the screen, three randomly drawn stimuli and one stimulus matching the sample. The bonobo was rewarded for selecting the matching stimulus, whereas a response to any of the other three stimuli was scored as incorrect and resulted in a black-out screen for 5 seconds. After the criterion of 80% correct choices within the last 20 trials was met, probe trials were introduced. Now, every block of ten trials included one probe trial, at a random location within these ten trials.

During a probe trial, instead of being presented a single sample stimulus, a bonobo was subjected to one of six sequences of two stimuli. Sequences were of the nature *AB*, where both stimuli were different and randomly drawn from the 20 test stimuli for each probe trial. After the second stimulus in the sequence disappeared, four stimuli from the common stimulus pool appeared. However, now both stimuli that occurred in the probe sequence were present, together with two non-matching stimuli. It was only possible to select one stimulus after a probe sequence, as all four stimuli disappeared after a stimulus had been selected. No responses were rewarded during probe trials. This way, we could measure if the bonobos selected the first or the second stimulus in the stimulus sequence.

We were interested in testing if different combinations of stimulus durations produced systematic patterns of choice as predicted by the trace memory model, which states that the duration of a stimulus will affect the intensity of the memory trace of that stimulus (see Fig 3 in [23]). Therefore, we varied the duration of both the first and the second stimulus during probe trials. For example, after a probe stimulus sequence *AB*, from a trace model a response to *A* could be expected with sequence durations such as *A*_long_*B*_short_, because this sequence would result in a stronger trace of *A* than *B*. The exact prediction from a trace model will depend on stimulus durations and the factor determining how quickly the trace memory decays. To compare responses by the bonobos with trace model predictions we varied durations of *A* and *B* systematically. In three of the probe sequences the first stimulus duration was held constant (*A* = 1s) and the second stimulus was presented at three durations (*B* = 0.5, 1.5, and 4.5s), and in the three other cases the first stimulus was presented at three durations (*A* = 0.5, 1.5, and 4.5s) and the second stimulus was held constant (*B* = 1.0s, see also Table 2). Each bonobo was subjected to 20 occurrences of the six probe sequences, totalling 120 probe trials.

### Analysis

The automated program used during experiments produced output-files automatically, and statistical analyses were performed with JASP 0.16.4.

## Results

### Bonobos

**Fig 1:**
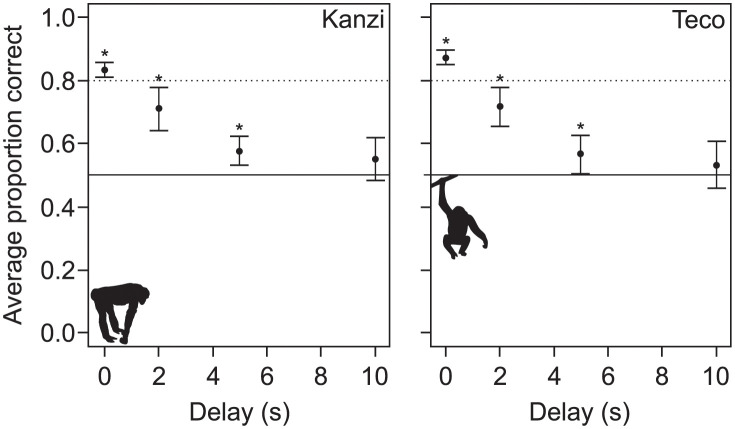
Delayed matching-to-sample. Average performance of two bonobos on delayed matching-to-sample at four different delays (error bars show 95% confidence intervals). Asterisks indicate results significantly above chance level.

The two bonobos learned the simultaneous matching-to-sample in 1691 and 1701 trials, and the zero-delay matching-to-sample after additional 120 and 198 trials, respectively (i.e. having reached the criterion of 80% correct choices over the last 20 trials). When testing memory performance in the delayed matching-to-sample task, both bonobos remembered stimuli better than chance for delays up to five seconds (average performance and p-values from binomial tests with the number of trials per duration, for Kanzi: 85% at 0 s (p<0.001, n = 866), 71% at 2 s (p<0.001, n = 190), 58% at 5 s (p = 0.001, 464), and 55% at 10 s (p = 0.16, n = 207), Teco: 88% at 0 s (p<0.001, n = 692), 72% at 2 s (p<0.001, n = 226), 57% at 5 s (p = 0.037, n = 267), and 53% at 10 s (p = 0.43, n = 194), and see Fig 1).

**Fig 2:**
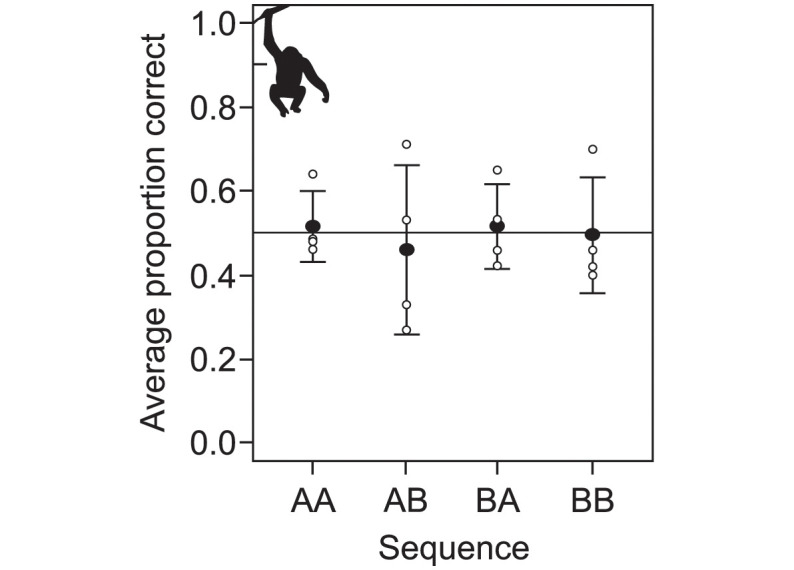
Performance on the last 120 trials in the sequence discrimination for bonobos. Filled circles show average proportion correct choices for the four stimulus sequences in the last block of sequence discrimination for bonobos. Open circles show individual data (n = 4, and error bars show standard deviation).

**Fig 3:**
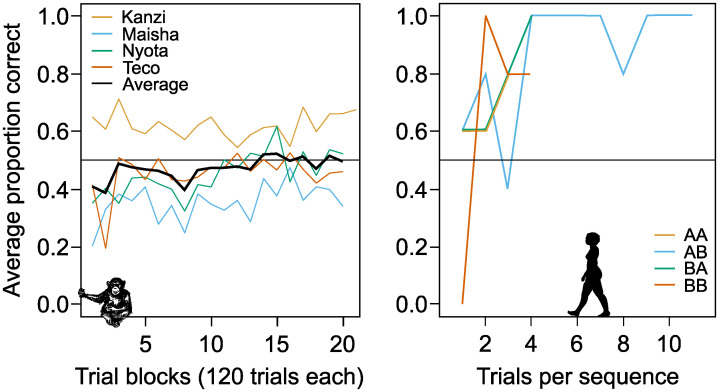
Sequence discrimination in bonobos and humans. Left panel shows the performance of bonobos (n = 4) throughout the stimulus sequences task, and the right panel shows average results for human subjects (n = 5) for the same task but shown per sequence. Note that bonobo performance was measured as the average proportion of correct trials for each block and that within each 120-trial block, 60 trials were *AB* trials, and the other 60 trials were either *AA*, *BA* or *BB* trials. Humans were subjected to the same proportion of stimulus sequences as the bonobos. In the right panel, lines end after criterion was met, that is 80% correct responses within the last 20 trials.

When examining the result from the sequence discrimination test we found that the four bonobos did not learn to tell the stimulus sequences apart with any precision, even after on average 2,370 trials. To see if performance improved at the end of the test we looked at performance over the final block of 120 trials. The average performance of four bonobos to learn to tell the four stimulus sequences apart was close to, or at, chance level (*AA*: 52% (±8 S.D.) correct choices, *AB*: 46% (±20 S.D.), *BA*: 52% (±10 S.D.), *BB*: 50% (±14 S.D.), and Fig 2). When looking at all trials performance did deviate from chance, both above and below 50%, but performance was never close to 80% correct for any of the bonobos on any of the four sequences (Table 1 and Fig 3).

**Table 1:** Performance in sequence discriminations of four bonobos. Values are percent correct with number of trials in brackets and p-value from a binomial test.

Subject	*AA*	*AB*	*BA*	*BB*
Kanzi	63(401), 0.001	63(1250), 0.001	61(421), 0.001	63(415), 0.001
Maisha	41(356), 0.001	31(1346), 0.001	44(305), 0.039	40(346), 0.001
Nyota	41(413), 0.001	49(1032), 0.73	40(427), 0.001	37(456), 0.001
Teco	47(354), 0.22	42(1199), 0.001	43(381), 0.008	45(379), 0.04

To test if the memory trace model could predict responses to stimulus sequences in bonobos, we first examined if the bonobos responded differently to the different probe types. Neither of the two bonobos responded differently than what was expected from chance levels (*χ*^2^-test for equality of distribution (between choosing *A* and *B* after each probe type): *χ*^2^ = 6, 48, p = 0.13 for Kanzi and *χ*^2^ = 5.06 p = 0.41 for Teco, see Table 2 for the number of responses to the six different stimulus sequences we used in the probe trials). However, their responding was not at random with respect to selecting one of the two matching stimuli (*A* or *B*) vs. the two non-matching stimuli. Both apes selected one of the stimuli from the sample sequence more often than the non-matching stimuli, as Kanzi selected either *A* or *B* in 116 of 120 probe trials (binomial test, testing the null hypothesis that selecting one matching stimulus (*A* or *B*) was equally likely as selecting one of the two non-matching stimuli, *p* < 0.001), and Teco selected either *A* or *B* in 94 of 120 probe trials (binomial test, *p* < 0.001). But, as their responses with respect to *A* and *B* did not deviate from chance level, we did not proceed with fitting data to the trace model.

**Table 2:** Duration (seconds) of the first and second stimuli, *A* and *B* respectively, of probe trial sequences during the trace test phase, and the number of responses for each probe trial type.

*A*	*B*	Kanzi, responses to *A* & *B*	Teco, responses to *A* & *B*
0.5	1.0	6, 14	4, 13
1.5	1.0	5, 15	4, 12
4.5	1.0	8, 11	4, 11
1.0	0.5	6, 13	7, 7
1.0	1.5	1, 18	3, 12
1.0	4.5	3, 16	3, 14

### Humans

Based on estimating performance in blocks of twenty trials, all five humans learned the sequence discrimination nearly immediately, with on average 79% correct choices after 22.4 trials (range 20–28 trials, individual binomial tests for five subjects: *p* = 0.036, *p* = 0.035, *p* = 0.012, *p* = 0.027, *p*<0.001, see Fig 3).

## Discussion

This study set out to test the hypothesis that memory for stimulus sequences is a cognitive divide between humans and other animals [23], because so far no data has been available for non-human great apes. This hypothesis states that non-human animals do not represent stimulus sequences faithfully, but as unstructured collections of memory traces. Our results corroborate two previous findings with respect to this hypothesis. First, in the delayed matching-to-sample test, bonobos’ memory for arbitrary single stimuli decays rapidly (Fig 1). This does not mean that bonobos and other animals cannot form other kinds of long-term memories [78] (reviewed in e.g. [34, 79, 80]), but it does mean that working memory for arbitrary stimuli in bonobos follows the same general pattern found in other non-human mammals and birds, in stark contrast to human working memory that can form long-term memories of arbitrary stimuli [46, 60]. Second, the sequence memory test showed that bonobos do not recognize and remember stimulus sequences with any precision (Fig 2), just like all other tested non-human mammals and birds [23]. When humans were subjected to the same sequence memory test it confirmed that humans recognize sequences, and the order of stimuli, with ease, as they learned to recognize all four stimulus sequences almost immediately (Fig 3).

It should be noted that our explicit test of the hypothesis that bonobos, like other animals [23], represent stimulus sequences as unstructured collections of memory traces was unsuccessful. The responses of the two bonobos did not vary systematically to what we expected from a trace memory-model. We expected there to be differences in responses depending on the relative duration of the first and second stimulus in the stimulus sequence probes, and can only conclude that further studies are needed to understand what causes the lack of faithful memory for stimulus sequences in bonobos.

A lack of memory for stimulus sequences has consequences for our understanding of animal cognition. If the order of perceived stimuli is not represented, and if this acts as a cognitive bottleneck for subsequent cognitive processes and behavior, then can we expect to find any information processing mechanisms that depend upon some exact ordering of information? For this reason, are for instance causal learning, language, episodic memory, and true imitation at all possible for non-human animals [34]? At the very least, these findings on the limits of memory for stimulus sequences (see also [23]) suggest that tentative cognitive bottlenecks and their consequences may have important consequences for understanding mental differences between humans and other animals.

Sequences are everywhere, and in animal communication individuals perceive sequences of information all the time. Song learning in birds is sequential by nature, and possible through genetic specializations [81]. Nevertheless, according to previous analyses [23] birds capable of learning sequences of song elements have not been found to represent stimulus sequences faithfully (e.g. starlings [7] and zebra finches [82]). The role of sequences, and order, of signals in animal communication is still not clear [83], and our results on memory limits in animals may prove useful for learning about the meaning and use of signals in the animal kingdom, for instance in fields such as auditory scene analysis [84], and receiver psychology [85].

As mentioned in the introduction, it has been a challenge to find distinct cognitive differences between humans and other animals. A recent review by Seed and Laland [5] concluded that “there are no traits present in humans and absent in other animals” that solely can explain the cognitive differences between humans and other animals. The fact that bonobos, the closest living relatives to humans, do not represent sequences of stimuli faithfully supports the hypothesis that memory for stimulus sequences is at least one part of basal cognitive differences between humans and other animals. To the best of our knowledge, no study to date has shown faithful memory for arbitrary stimulus sequences in a non-human animal, whereas such memory is key for human everyday life when we speak and listen, prepare and cook meals, and create and maintain personal systems of beliefs. This idea forms the basis for a recent book where Enquist, Ghirlanda, and Lind, explore the human evolutionary transition and argue that memory for stimulus sequences, together with sequential processing of information may be important aspects of a cognitive and cultural divide between humans and all other extant animals [6].

## Conclusion

Two observations in this study support the idea that bonobos lack a faithful memory for stimulus sequences. First, bonobos, like other non-human animals, forget arbitrary stimuli within a short time span, suggesting they do not form long-lasting memories for arbitrary stimuli. Second, it was difficult for bonobos to learn to tell short stimulus sequences apart. These patterns match results found in all other tested non-human animals on both arbitrary single stimuli [60] and sequences of stimuli [23]. This study highlights differences between humans’ and other animals’ general-purpose memory systems as tentative causes for the observed cognitive and cultural divide between humans and other animals.

## Supporting information

S1 Appendix Supporting information containing screenshots. This file contains representative screenshots from the different parts of the study.
(PDF)

S1 Dataset Datasets and readme-file. This file contains data from all included tests and an explanatory readme-file.
(ZIP)
