# Supplementary material for: A test of memory for stimulus sequences in great apes
Source: PLoS One. 2023 Sep 6;18(9):e0290546. doi: 10.1371/journal.pone.0290546 (PMC10482264; doi:10.1371/journal.pone.0290546)
Supplement: S1 Appendix — This file contains representative screenshots from the different parts of the study. (PDF) [file pone.0290546.s001.pdf]

Supporting information for “A test of memory for stimulus sequences in great apes”

Johan Lind, Vera Vinken, Markus Jonsson,
Stefano Ghirlanda & Magnus Enquist

August 14, 2023

1 Screenshots from different parts of the study

Below are representative screenshots from the different parts of the study. For descriptions of procedure, see “Materials and methods” in the manuscript.

1.1 Delayed matching-to-sample in bonobos

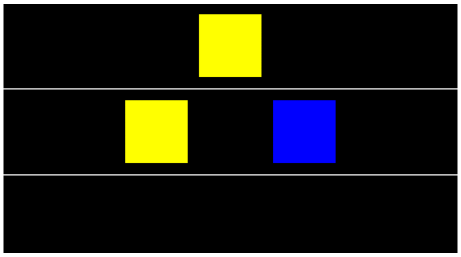

Figure 1: Screenshot from simultaneous matching-to-sample to show all elements on the screen. During delayed matching-to-sample, the screen turned black during the delay, and after the delay only the two bottom, matching and non-matching, stimuli appeared.

1.2 Sequence discrimination in bonobos

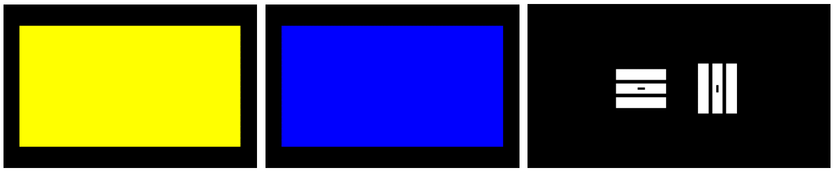

Figure 2: From left to right are screenshots for yellow stimulus, blue stimulus, and the two response buttons.

1.3 Sequence discrimination in humans

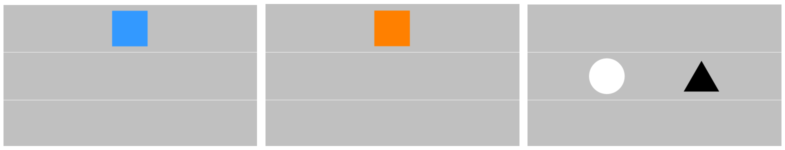

Figure 3: From left to right are screenshots for blue stimulus, orange stimulus, and the two response buttons.

Human subjects received no food rewards after correct responses. To provide information about correct choices in the sequence discrimination in humans, happy and sad faces were used instead of food rewards and lack of food reward.

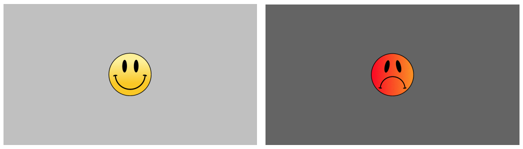

Figure 4: To the left, a happy face that was presented after a correct response, and to the right a sad face that was presented after an incorrect response.

1.4 Trace model test in bonobos

In the trace model test, a pool of twenty stimuli were used in a modified zero-delay matching-to-sample test.

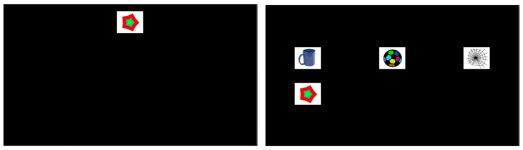

Figure 5: To the left, a sample stimulus is presented on the screen, and to the right is the screen with one matching stimulus together with three non-matching stimuli.

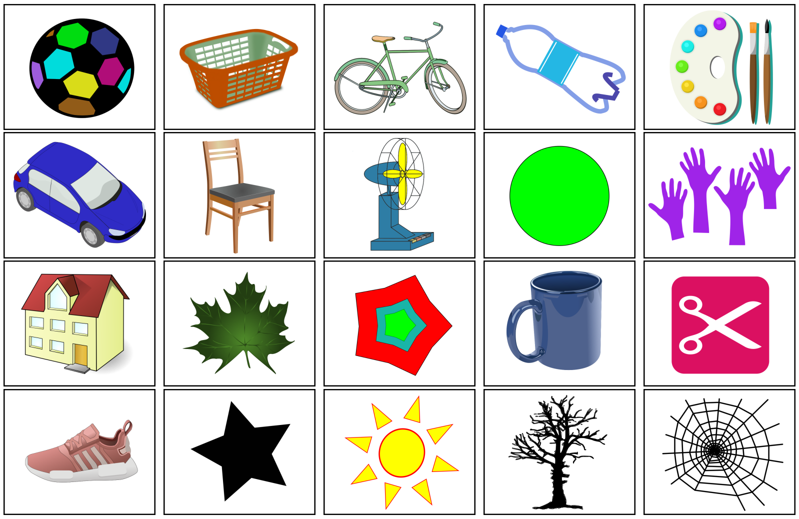

Figure 6: The stimulus pool in the trace model test consisted of these 20 stimuli. For the relative size of these stimuli on screen, see figure 5.
